# 7,7-Dimethyl-4a-(3-methyl-2-buten­yl)-2-oxo-4a,5,6,7-tetra­hydro-2*H*-chromen-4-yl benzoate

**DOI:** 10.1107/S1600536809030918

**Published:** 2009-08-12

**Authors:** Katrin Möws, Markus Schürmann, Hans Preut, Bernd Plietker

**Affiliations:** a Fakultät Chemie, Technische Universität Dortmund, Otto-Hahn-Strasse 6, 44221 Dortmund, Germany; b Institut für Organische Chemie, Fakultät Chemie, Universität Stuttgart, Pfaffenwaldring 55, 70569 Stuttgart, Germany

## Abstract

An intra­molecular Claisen-like cyclization of ethyl 2-acet­oxy-4,4-dimethyl-1-(3-methyl­but-2-en­yl)cyclo­hex-2-enecarboxylate followed by dialkyl­ation yielded the bicyclic title compound, C_23_H_26_O_4_. In both of the fused six-membered rings exist fragments of four atoms which are planar, whereas the remaining two atoms deviate by up to 0.682 (3) Å on one side of the plane of the ring containing an O atom and by up to 0.415 (3) Å on opposite sides of the other ring. The dihedral anglebetween the planar fragments of the six-membered rings is 41.76 (10)°

## Related literature

For literature related to the synthesis, see: Ciochina & Grossman (2006 ▶).
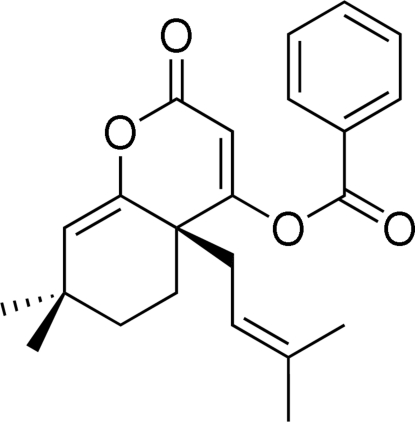


## Experimental

### 

#### Crystal data

C_23_H_26_O_4_
*M* *_r_* = 366.44
Triclinic, 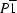

*a* = 9.559 (4) Å
*b* = 10.201 (5) Å
*c* = 11.087 (5) Å
α = 69.23 (2)°
β = 83.649 (17)°
γ = 74.823 (17)°
*V* = 975.4 (8) Å^3^
*Z* = 2
Mo *K*α radiation
μ = 0.08 mm^−1^
*T* = 173 K
0.45 × 0.40 × 0.20 mm

#### Data collection

Nonius KappaCCD diffractometer
Absorption correction: none
12665 measured reflections
3547 independent reflections
1691 reflections with *I* > 2σ(*I*)
*R*_int_ = 0.065

#### Refinement

*R*[*F* ^2^ > 2σ(*F* ^2^)] = 0.043
*wR*(*F* ^2^) = 0.067
*S* = 0.81
3547 reflections
248 parameters
H-atom parameters constrained
Δρ_max_ = 0.17 e Å^−3^
Δρ_min_ = −0.14 e Å^−3^

### 

Data collection: *COLLECT* (Nonius, 1998 ▶); cell refinement: *DENZO* and *SCALEPACK* (Otwinowski & Minor, 1997 ▶); data reduction: *DENZO* and *SCALEPACK*; program(s) used to solve structure: *SHELXS97* (Sheldrick, 2008 ▶); program(s) used to refine structure: *SHELXL97* (Sheldrick, 2008 ▶); molecular graphics: *SHELXTL-Plus* (Sheldrick, 2008 ▶); software used to prepare material for publication: *SHELXL97* and *PLATON* (Spek, 2009 ▶).

## Supplementary Material

Crystal structure: contains datablocks I, global. DOI: 10.1107/S1600536809030918/hg2544sup1.cif

Structure factors: contains datablocks I. DOI: 10.1107/S1600536809030918/hg2544Isup2.hkl

Additional supplementary materials:  crystallographic information; 3D view; checkCIF report

## Figures and Tables

**Fig. 1.**
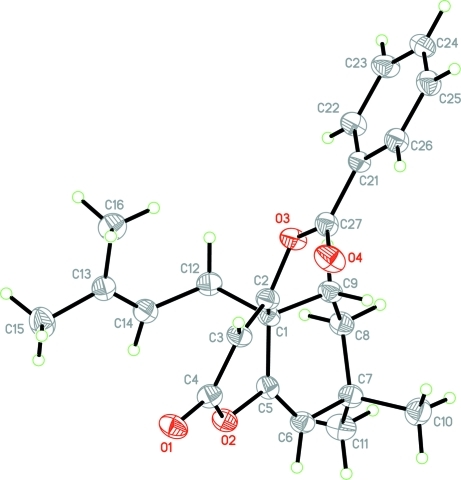
: The asymmetric unit of the title compound showing the labelling of all non-H atoms. Displacement ellipsoids are shown at the 30% probability level. Of the two disordered positions C35 and C35' only one is shown.

## supplementary crystallographic information

### Experimental

Diethylether was dryed over sodium. All other solvents and reagents were commercially available and used as received. Flash-chromatography was done on silicagel 60 (230–400 mesh) using head pressure by means of compressed air. Infrared spectra (IR) were recorded as a thin film between KBr-plates. The instrument used was a Bruker IFS 66 F T—IR spectrophotometer. GC—MS spectra were recorded on a Finnigan Polaris GCQ spectrometer. Proton (^1^H NMR, 500 MHz) and carbon (^13^C NMR, 125 MHz) nuclear magnetic resonance spectra were recorded in chloroform(d-1) and referenced to the solvent signal. The instrument used was a Bruker DRX 500. The multiplicities of the signals are given as s (singlet), d (doublet), t (triplet), and m (multiplet).

HMDS (468 µ*L*, 2.25 mmol) was dissolved in diethylether (3 ml) at 273 K. Butyllithium (1.3 ml, 2.03 mmol, 1.6 *M* in hexane) was added at that temperature and the mixture was stirred for 15 minutes. After cooling to 195 K a suspension of CuI (216 mg, 1.13 mmol) and ethyl 2-acetoxy-4,4-dimethyl-1-(3-methylbut-2-enyl)cyclohex-2-enecarboxylate (348 mg, 1.13 mmol) in diethylether (3 ml) was added. The mixture was stirred for 2 h. Benzoyl chloride (88 µl, 2.25 mmol) was added dropwise and the mixture was stirred for 5 days at room temperature. An aqueous Seignette salt-solution was added. Phase were separated and the aqueous layer was extracted with Et_2_O (3 *x* 10 ml). The combined organic layers were dried over Na_2_SO_4_ and concentrated in vacuum (Ciochina & Grossman, 2006). The crude product was purified *via* column chromatography (10:1 i-hexane/ethyl acetate). The product was obtained in 12% yield (50 mg, 0.136 mmol). The purified product was crystallized by slow evaporation of a mixture of diethylether and i-hexane.

*R*_f_: 0.22 (i-hexane/ethyl acetate 10:1), mp: 387 K, ^1^H NMR (400 MHz, CDCl_3_): δ (p.p.m.) = 8.08–7.50 (m, 5H, CH), 6.33 (s, 1H, CH), 5.28 (s, 1H, CH), 5.21 (m, 1H, CH), 2.62 (dd, J = 13.9, 7.7 Hz, 1H, CH_2_), 2.43 (dd, J = 13.9, 8.5 Hz, 1H, CH_2_), 2.06 (dt, J = 13.4, 3.5 Hz, 1H, CH_2_), 1.72 (dt, J = 13.9, 3.0 Hz, 1H, CH_2_), 1.65 (s, 3H, CH_3_), 1.59 (s, 3H, CH_3_), 1.57–1.52 (m, 1H, CH_2_), 1.10 (s, 3H, CH_3_), 1.03 (s, 3H, CH_3_). ^13^C NMR (125 MHz, CDCl_3_): δ (p.p.m.) = 168.7, 162.9, 162.4, 147.8, 136.8, 134.5, 130.4, 129.0, 120.3, 118.3, 105.1, 42.3, 36.7, 32.8, 31.9, 31.2, 26.5, 26.1. IR (film): ν (cm^-1^) = 2966 (*m*), 2945 (*m*), 2919 (*m*), 2863 (*m*), 1759 (*s*), 1736 (*s*), 1673 (*m*), 1636 (*m*), 1452 (*m*), 1366 (*m*), 1234 (*s*), 1145 (*s*), 1077 (*s*), 1065 (*s*), 1020 (*m*). MS (EI, 70 eV): m / *z* (%) = 366 (10) [*M*+], 351 (1) [C_22_H_23_O_4_^+^], 311 (1) [C_19_H_19_O_4_^+^], 261 (100) [C_16_H_21_O_3_^+^], 245 (5) [C_16_H_21_O_2_^+^], 219 (2) [C_13_H_15_O_3_^+^], 121 (1) [C_7_H_5_O_2_^+^], 105 (99) [C_7_H_5_O^+^], 77 (26) [C_6_H_5_^+^]. LRMS (FAB^+^LR,*C*_23_H_26_O_4_) calc. [(*M*+H)^+^]: 367.18; found: 367.02.

### Refinement

H atoms were placed in calculated positions, with C—H = 0.95–0.99 Å and were refined as riding, with *U*_iso_= 1.5*U*_eq_ for methyl and 1.2*U*_eq_ for others; the methyl were allowed to rotate but not to tip.

### Crystal data

**Table:**

C_23_H_26_O_4_	*Z* = 2
*M**_r_* = 366.44	*F*(000) = 392
Triclinic, *P*1	*D*_x_ = 1.248 Mg m^−^^3^
Hall symbol: -P 1	Mo *K*α radiation, λ = 0.71073 Å
*a* = 9.559 (4) Å	Cell parameters from 12656 reflections
*b* = 10.201 (5) Å	θ = 2.9–25.3°
*c* = 11.087 (5) Å	µ = 0.08 mm^−^^1^
α = 69.23 (2)°	*T* = 173 K
β = 83.649 (17)°	Block, colourless
γ = 74.823 (17)°	0.45 × 0.40 × 0.20 mm
*V* = 975.4 (8) Å^3^	

### Data collection

**Table:**

Nonius KappaCCD diffractometer	1691 reflections with *I* > 2σ(*I*)
Radiation source: fine-focus sealed tube	*R*_int_ = 0.065
graphite	θ_max_ = 25.3°, θ_min_ = 2.9°
Detector resolution: 19 vertical, 18 horizontal pixels mm^-1^	*h* = −11→11
239 frames via ω–rotation (Δω=2°) and two times 20 s per frame (four sets at different κ–angles) scans	*k* = −11→12
12665 measured reflections	*l* = −13→13
3547 independent reflections	

### Refinement

**Table:**

Refinement on *F*^2^	Primary atom site location: structure-invariant direct methods
Least-squares matrix: full	Secondary atom site location: difference Fourier map
*R*[*F*^2^ > 2σ(*F*^2^)] = 0.043	Hydrogen site location: inferred from neighbouring sites
*wR*(*F*^2^) = 0.067	H-atom parameters constrained
*S* = 0.81	*w* = 1/[σ^2^(*F*_o_^2^)]
3547 reflections	(Δ/σ)_max_ < 0.001
248 parameters	Δρ_max_ = 0.17 e Å^−^^3^
0 restraints	Δρ_min_ = −0.14 e Å^−^^3^

### Special details

**Table:**

Geometry. All e.s.d.'s (except the e.s.d. in the dihedral angle between two l.s. planes) are estimated using the full covariance matrix. The cell e.s.d.'s are taken into account individually in the estimation of e.s.d.'s in distances, angles and torsion angles; correlations between e.s.d.'s in cell parameters are only used when they are defined by crystal symmetry. An approximate (isotropic) treatment of cell e.s.d.'s is used for estimating e.s.d.'s involving l.s. planes.
Refinement. Refinement of *F*^2^ against ALL reflections. The weighted *R*-factor *wR* and goodness of fit *S* are based on *F*^2^, conventional *R*-factors *R* are based on *F*, with *F* set to zero for negative *F*^2^. The threshold expression of *F*^2^ > σ(*F*^2^) is used only for calculating *R*-factors(gt) *etc*. and is not relevant to the choice of reflections for refinement. *R*-factors based on *F*^2^ are statistically about twice as large as those based on *F*, and *R*- factors based on ALL data will be even larger.

### Fractional atomic coordinates and isotropic or equivalent isotropic displacement parameters (Å^2^)

**Table:**

	*x*	*y*	*z*	*U*_iso_*/*U*_eq_	
O1	0.57097 (14)	0.80118 (14)	−0.03317 (12)	0.0520 (4)	
O2	0.46540 (13)	0.63064 (14)	0.09192 (11)	0.0425 (4)	
O3	0.14946 (14)	0.93092 (13)	0.21014 (11)	0.0436 (4)	
O4	0.08624 (14)	1.10136 (14)	0.01484 (13)	0.0592 (4)	
C1	0.2779 (2)	0.6848 (2)	0.25269 (17)	0.0372 (5)	
C2	0.2597 (2)	0.8390 (2)	0.16647 (17)	0.0386 (5)	
C3	0.3498 (2)	0.8812 (2)	0.06716 (17)	0.0420 (5)	
H3A	0.3364	0.9805	0.0163	0.050*	
C4	0.4696 (2)	0.7729 (2)	0.03743 (18)	0.0424 (6)	
C5	0.3483 (2)	0.5906 (2)	0.17409 (17)	0.0374 (5)	
C6	0.3123 (2)	0.4741 (2)	0.17565 (16)	0.0403 (5)	
H6A	0.3664	0.4240	0.1214	0.048*	
C7	0.1911 (2)	0.4141 (2)	0.25740 (17)	0.0427 (5)	
C8	0.1416 (2)	0.48837 (19)	0.35812 (17)	0.0438 (5)	
H8A	0.0457	0.4717	0.3940	0.053*	
H8B	0.2110	0.4442	0.4297	0.053*	
C9	0.1305 (2)	0.65023 (19)	0.30311 (17)	0.0423 (5)	
H9A	0.0926	0.6932	0.3710	0.051*	
H9B	0.0608	0.6948	0.2317	0.051*	
C10	0.0654 (2)	0.4414 (2)	0.16994 (17)	0.0564 (6)	
H10A	0.1009	0.4002	0.1016	0.085*	
H10B	0.0254	0.5456	0.1311	0.085*	
H10C	−0.0104	0.3960	0.2212	0.085*	
C11	0.2449 (2)	0.25092 (19)	0.32494 (17)	0.0554 (6)	
H11A	0.2777	0.2040	0.2601	0.083*	
H11B	0.1657	0.2123	0.3771	0.083*	
H11C	0.3256	0.2321	0.3808	0.083*	
C12	0.3724 (2)	0.6598 (2)	0.36940 (16)	0.0435 (5)	
H12A	0.3274	0.7340	0.4095	0.052*	
H12B	0.3699	0.5644	0.4347	0.052*	
C13	0.5899 (2)	0.7717 (2)	0.32049 (17)	0.0432 (5)	
C14	0.5284 (2)	0.6650 (2)	0.33670 (16)	0.0437 (5)	
H14A	0.5908	0.5802	0.3264	0.052*	
C15	0.7482 (2)	0.7596 (2)	0.28583 (18)	0.0613 (7)	
H15A	0.7934	0.6623	0.2839	0.092*	
H15B	0.7957	0.7777	0.3503	0.092*	
H15C	0.7589	0.8308	0.2007	0.092*	
C16	0.5118 (2)	0.9153 (2)	0.33373 (17)	0.0561 (6)	
H16A	0.4091	0.9170	0.3540	0.084*	
H16B	0.5209	0.9931	0.2525	0.084*	
H16C	0.5548	0.9291	0.4033	0.084*	
C21	−0.0634 (2)	1.1136 (2)	0.19952 (18)	0.0364 (5)	
C22	−0.0832 (2)	1.0524 (2)	0.33214 (17)	0.0460 (6)	
H22A	−0.0149	0.9691	0.3809	0.055*	
C23	−0.2037 (2)	1.1143 (2)	0.39196 (19)	0.0520 (6)	
H23A	−0.2180	1.0729	0.4824	0.062*	
C24	−0.3031 (2)	1.2347 (2)	0.3223 (2)	0.0518 (6)	
H24A	−0.3854	1.2758	0.3649	0.062*	
C25	−0.2839 (2)	1.2966 (2)	0.19050 (19)	0.0496 (6)	
H25A	−0.3526	1.3798	0.1421	0.059*	
C26	−0.1632 (2)	1.2356 (2)	0.13033 (18)	0.0445 (5)	
H26A	−0.1487	1.2781	0.0400	0.053*	
C27	0.0632 (2)	1.0537 (2)	0.1279 (2)	0.0422 (5)	

### Atomic displacement parameters (Å^2^)

**Table:**

	*U*^11^	*U*^22^	*U*^33^	*U*^12^	*U*^13^	*U*^23^
O1	0.0492 (10)	0.0545 (10)	0.0489 (9)	−0.0150 (8)	0.0184 (8)	−0.0175 (8)
O2	0.0430 (9)	0.0443 (9)	0.0372 (8)	−0.0111 (8)	0.0074 (7)	−0.0122 (7)
O3	0.0457 (9)	0.0418 (9)	0.0353 (8)	0.0002 (7)	0.0020 (7)	−0.0118 (7)
O4	0.0581 (11)	0.0648 (11)	0.0345 (9)	−0.0001 (8)	0.0044 (8)	−0.0049 (8)
C1	0.0352 (13)	0.0413 (13)	0.0327 (12)	−0.0075 (11)	0.0046 (10)	−0.0124 (10)
C2	0.0388 (14)	0.0386 (14)	0.0359 (12)	−0.0029 (11)	−0.0013 (11)	−0.0141 (11)
C3	0.0446 (14)	0.0410 (13)	0.0367 (12)	−0.0068 (11)	0.0022 (11)	−0.0120 (10)
C4	0.0491 (16)	0.0439 (15)	0.0321 (13)	−0.0131 (13)	0.0013 (11)	−0.0097 (11)
C5	0.0363 (14)	0.0414 (14)	0.0293 (12)	−0.0099 (11)	0.0023 (10)	−0.0062 (10)
C6	0.0432 (14)	0.0431 (14)	0.0326 (12)	−0.0082 (12)	0.0032 (10)	−0.0131 (10)
C7	0.0450 (14)	0.0433 (14)	0.0379 (12)	−0.0109 (12)	0.0019 (11)	−0.0124 (11)
C8	0.0441 (14)	0.0475 (14)	0.0387 (12)	−0.0137 (11)	0.0062 (10)	−0.0133 (11)
C9	0.0411 (14)	0.0486 (14)	0.0352 (12)	−0.0078 (11)	0.0043 (10)	−0.0154 (10)
C10	0.0586 (16)	0.0652 (16)	0.0495 (14)	−0.0228 (13)	0.0018 (13)	−0.0197 (12)
C11	0.0642 (16)	0.0468 (14)	0.0504 (14)	−0.0153 (13)	0.0086 (12)	−0.0122 (11)
C12	0.0496 (15)	0.0458 (14)	0.0330 (12)	−0.0120 (12)	0.0012 (11)	−0.0110 (10)
C13	0.0446 (15)	0.0510 (15)	0.0308 (12)	−0.0122 (13)	−0.0014 (10)	−0.0092 (11)
C14	0.0449 (15)	0.0474 (14)	0.0327 (12)	−0.0048 (12)	−0.0036 (11)	−0.0099 (11)
C15	0.0491 (16)	0.0791 (18)	0.0491 (14)	−0.0152 (13)	−0.0003 (12)	−0.0141 (12)
C16	0.0627 (16)	0.0566 (15)	0.0473 (13)	−0.0165 (13)	0.0000 (12)	−0.0143 (11)
C21	0.0355 (13)	0.0374 (13)	0.0352 (12)	−0.0069 (11)	0.0019 (10)	−0.0133 (10)
C22	0.0450 (15)	0.0465 (14)	0.0385 (13)	−0.0026 (12)	−0.0001 (11)	−0.0110 (11)
C23	0.0543 (16)	0.0579 (16)	0.0393 (13)	−0.0048 (13)	0.0042 (12)	−0.0188 (12)
C24	0.0419 (15)	0.0588 (16)	0.0540 (15)	−0.0029 (13)	0.0039 (12)	−0.0264 (13)
C25	0.0428 (15)	0.0520 (15)	0.0480 (15)	−0.0006 (12)	−0.0073 (12)	−0.0158 (12)
C26	0.0432 (14)	0.0456 (14)	0.0400 (12)	−0.0055 (12)	−0.0041 (11)	−0.0115 (11)
C27	0.0396 (14)	0.0412 (14)	0.0410 (13)	−0.0042 (11)	−0.0052 (12)	−0.0110 (12)

### Geometric parameters (Å, °)

**Table:**

O1—C4	1.206 (2)	C11—H11B	0.9800
O2—C4	1.369 (2)	C11—H11C	0.9800
O2—C5	1.414 (2)	C12—C14	1.505 (2)
O3—C27	1.377 (2)	C12—H12A	0.9900
O3—C2	1.3843 (19)	C12—H12B	0.9900
O4—C27	1.191 (2)	C13—C14	1.317 (2)
C1—C2	1.498 (2)	C13—C15	1.504 (2)
C1—C5	1.504 (2)	C13—C16	1.510 (2)
C1—C9	1.539 (2)	C14—H14A	0.9500
C1—C12	1.568 (2)	C15—H15A	0.9800
C2—C3	1.337 (2)	C15—H15B	0.9800
C3—C4	1.468 (2)	C15—H15C	0.9800
C3—H3A	0.9500	C16—H16A	0.9800
C5—C6	1.316 (2)	C16—H16B	0.9800
C6—C7	1.511 (2)	C16—H16C	0.9800
C6—H6A	0.9500	C21—C26	1.382 (2)
C7—C8	1.530 (2)	C21—C22	1.392 (2)
C7—C11	1.532 (2)	C21—C27	1.487 (2)
C7—C10	1.534 (2)	C22—C23	1.383 (2)
C8—C9	1.522 (2)	C22—H22A	0.9500
C8—H8A	0.9900	C23—C24	1.373 (2)
C8—H8B	0.9900	C23—H23A	0.9500
C9—H9A	0.9900	C24—C25	1.384 (2)
C9—H9B	0.9900	C24—H24A	0.9500
C10—H10A	0.9800	C25—C26	1.383 (2)
C10—H10B	0.9800	C25—H25A	0.9500
C10—H10C	0.9800	C26—H26A	0.9500
C11—H11A	0.9800		
			
C4—O2—C5	120.47 (15)	H11A—C11—H11B	109.5
C27—O3—C2	122.64 (14)	C7—C11—H11C	109.5
C2—C1—C5	107.80 (15)	H11A—C11—H11C	109.5
C2—C1—C9	111.32 (16)	H11B—C11—H11C	109.5
C5—C1—C9	107.82 (16)	C14—C12—C1	115.38 (15)
C2—C1—C12	107.95 (16)	C14—C12—H12A	108.4
C5—C1—C12	112.42 (15)	C1—C12—H12A	108.4
C9—C1—C12	109.54 (14)	C14—C12—H12B	108.4
C3—C2—O3	125.00 (17)	C1—C12—H12B	108.4
C3—C2—C1	122.81 (17)	H12A—C12—H12B	107.5
O3—C2—C1	111.89 (16)	C14—C13—C15	121.7 (2)
C2—C3—C4	119.40 (18)	C14—C13—C16	124.62 (19)
C2—C3—H3A	120.3	C15—C13—C16	113.69 (19)
C4—C3—H3A	120.3	C13—C14—C12	128.56 (19)
O1—C4—O2	117.76 (19)	C13—C14—H14A	115.7
O1—C4—C3	124.16 (19)	C12—C14—H14A	115.7
O2—C4—C3	118.08 (18)	C13—C15—H15A	109.5
C6—C5—O2	117.13 (17)	C13—C15—H15B	109.5
C6—C5—C1	126.20 (18)	H15A—C15—H15B	109.5
O2—C5—C1	116.66 (17)	C13—C15—H15C	109.5
C5—C6—C7	124.84 (18)	H15A—C15—H15C	109.5
C5—C6—H6A	117.6	H15B—C15—H15C	109.5
C7—C6—H6A	117.6	C13—C16—H16A	109.5
C6—C7—C8	109.01 (16)	C13—C16—H16B	109.5
C6—C7—C11	109.75 (16)	H16A—C16—H16B	109.5
C8—C7—C11	109.79 (16)	C13—C16—H16C	109.5
C6—C7—C10	108.99 (15)	H16A—C16—H16C	109.5
C8—C7—C10	110.54 (16)	H16B—C16—H16C	109.5
C11—C7—C10	108.74 (17)	C26—C21—C22	119.58 (18)
C9—C8—C7	112.82 (15)	C26—C21—C27	117.90 (17)
C9—C8—H8A	109.0	C22—C21—C27	122.51 (18)
C7—C8—H8A	109.0	C23—C22—C21	119.15 (19)
C9—C8—H8B	109.0	C23—C22—H22A	120.4
C7—C8—H8B	109.0	C21—C22—H22A	120.4
H8A—C8—H8B	107.8	C24—C23—C22	120.90 (19)
C8—C9—C1	112.10 (15)	C24—C23—H23A	119.6
C8—C9—H9A	109.2	C22—C23—H23A	119.6
C1—C9—H9A	109.2	C23—C24—C25	120.33 (19)
C8—C9—H9B	109.2	C23—C24—H24A	119.8
C1—C9—H9B	109.2	C25—C24—H24A	119.8
H9A—C9—H9B	107.9	C26—C25—C24	119.00 (19)
C7—C10—H10A	109.5	C26—C25—H25A	120.5
C7—C10—H10B	109.5	C24—C25—H25A	120.5
H10A—C10—H10B	109.5	C21—C26—C25	121.03 (18)
C7—C10—H10C	109.5	C21—C26—H26A	119.5
H10A—C10—H10C	109.5	C25—C26—H26A	119.5
H10B—C10—H10C	109.5	O4—C27—O3	123.95 (18)
C7—C11—H11A	109.5	O4—C27—C21	125.59 (19)
C7—C11—H11B	109.5	O3—C27—C21	110.43 (17)
			
C27—O3—C2—C3	39.7 (3)	C6—C7—C8—C9	42.2 (2)
C27—O3—C2—C1	−146.56 (16)	C11—C7—C8—C9	162.48 (16)
C5—C1—C2—C3	−29.9 (3)	C10—C7—C8—C9	−77.6 (2)
C9—C1—C2—C3	−147.98 (18)	C7—C8—C9—C1	−62.2 (2)
C12—C1—C2—C3	91.8 (2)	C2—C1—C9—C8	163.77 (15)
C5—C1—C2—O3	156.15 (15)	C5—C1—C9—C8	45.7 (2)
C9—C1—C2—O3	38.1 (2)	C12—C1—C9—C8	−76.91 (19)
C12—C1—C2—O3	−82.17 (19)	C2—C1—C12—C14	−68.5 (2)
O3—C2—C3—C4	175.08 (16)	C5—C1—C12—C14	50.2 (2)
C1—C2—C3—C4	2.0 (3)	C9—C1—C12—C14	170.09 (16)
C5—O2—C4—O1	−179.08 (16)	C15—C13—C14—C12	−179.07 (16)
C5—O2—C4—C3	0.1 (2)	C16—C13—C14—C12	0.7 (3)
C2—C3—C4—O1	−165.68 (19)	C1—C12—C14—C13	100.6 (2)
C2—C3—C4—O2	15.2 (3)	C26—C21—C22—C23	0.6 (3)
C4—O2—C5—C6	149.39 (17)	C27—C21—C22—C23	180.00 (18)
C4—O2—C5—C1	−31.3 (2)	C21—C22—C23—C24	−0.1 (3)
C2—C1—C5—C6	−137.1 (2)	C22—C23—C24—C25	−0.2 (3)
C9—C1—C5—C6	−16.8 (3)	C23—C24—C25—C26	−0.1 (3)
C12—C1—C5—C6	104.0 (2)	C22—C21—C26—C25	−1.0 (3)
C2—C1—C5—O2	43.7 (2)	C27—C21—C26—C25	179.64 (17)
C9—C1—C5—O2	163.97 (14)	C24—C25—C26—C21	0.7 (3)
C12—C1—C5—O2	−75.2 (2)	C2—O3—C27—O4	−9.8 (3)
O2—C5—C6—C7	179.69 (15)	C2—O3—C27—C21	168.29 (15)
C1—C5—C6—C7	0.5 (3)	C26—C21—C27—O4	−0.4 (3)
C5—C6—C7—C8	−12.7 (3)	C22—C21—C27—O4	−179.8 (2)
C5—C6—C7—C11	−133.0 (2)	C26—C21—C27—O3	−178.48 (17)
C5—C6—C7—C10	108.0 (2)	C22—C21—C27—O3	2.2 (3)
